# Performance improvement in electrospun InGaZnO nanofibres field-effect-transistors using low thermal budget microwave calcination and Ar/O_2_ mixed-plasma surface treatment

**DOI:** 10.1038/s41598-020-60637-8

**Published:** 2020-02-27

**Authors:** Seong-Kun Cho, Won-Ju Cho

**Affiliations:** Department of Electronic Materials Engineering, Kwangwoon University, Gwangun-ro 20, Nowon-gu, Seoul, 01897 Republic of Korea

**Keywords:** Nanowires, Electronic devices

## Abstract

In this study, we present a low thermal budget microwave annealing (MWA) method for calcination of electrospun In-Ga-ZnO (IGZO) nanofibres and demonstrate an improvement in the performance of IGZO nanofibre field-effect transistors (FETs) by Ar/O_2_ mixed-plasma surface treatment. The IGZO nanofibres were fabricated by electrospinning method and calcined using MWA method. This process allowed for a significant reduction in the heat treatment temperature and time. Subsequently, plasma surface treatment using various ratios of Ar/O_2_ gas mixtures was carried out. The surface morphology and chemical composition of MWA-calcined and plasma-treated IGZO nanofibres were studied by SEM and XPS analysis. In order to investigate the effects of MWA calcination combined with Ar/O_2_ mixed-plasma treatment on the electrical properties and the reliability of nanofibres-based transistors, IGZO nanofibres FETs were fabricated and applied to resistor-loaded inverters. Our results show that the O_2_ plasma treatment significantly improves the performance of IGZO nanofibres FETs and the resistor-loaded inverters based on IGZO nanofibres FETs, whereas Ar plasma treatment degrades the performance of these devices. The instability tests using positive bias temperature stress (PBTS) and negative bias temperature stress (NBTS) revealed that the O_2_ plasma treatment contributed to the stability of IGZO nanofibres FETs. Our results suggest that the MWA calcination combined with the Ar/O_2_ mixed-plasma surface treatment is a promising technique for the fabrication of high performance IGZO nanofibres FETs with low thermal budget processes.

## Introduction

Recently, the application of amorphous oxide semiconductors (AOS) as backplanes for the thin film transistors (TFTs) of active matrix liquid crystal displays (AMLCDs) and active matrix organic light emitting diode displays (AMOLEDs) has been an actively researched topic owing to its advantages over the currently existing technology^1,2^. Especially, amorphous indium gallium zinc oxide (*a*-IGZO) TFTs exhibit higher mobility than amorphous silicon (*a*-Si:H) TFTs, and they possess a superior uniformity compared with the polycrystalline silicon (poly-Si) TFTs because of their amorphous structure^3–5^. In addition, *a*-IGZO has a wide band gap and high transmittance in the visible region, which makes it easy to access transparent optoelectronics^6,7^. Despite these advantages, achieving a desired flexibility and stretchability in *a*-IGZO films still remains challenging. In addition, IGZO deposited by vacuum equipment, such as radio frequency (RF) magnetron sputtering, chemical vapor deposition (CVD) or atomic-layer-deposited (ALD), requires expensive equipment, and is disadvantageous for long process time and large area deposition. Recent intensive efforts have been focussed on the fabrication of IGZO nanofibres for their applications in the next-generation electronics, which are more flexible and stretchable^8–10^. Electrospinning is one of the most commonly used manufacturing methods of nanofibres owing to its low manufacturing cost and simple procedure that can be carried out in absence of vacuum, since it is solution process based^9–11^. Additionally, compared to vacuum equipment such as sputters, it is advantageous to easily control the composition ratio of IGZO and to deposit large-area at room temperature^11^. In particular, electrospun semiconductor nanofibres provide flexibility in the design of channel materials, efficient modulation of carriers in the channel, and ease of scale-up to large area devices. While IGZO has excellent electrical properties among many oxide semiconductors, electrospun IGZO nanofibres are attractive materials with high flexibility and large specific surface area in one-dimensional (1D) form^12^. However, electrospun IGZO nanofibres require high temperature calcination annealing to vaporize the polymer matrix and high temperature post deposition annealing (PDA) to remove defects in the metal oxides and to improve the electrical properties^9,10,13^. These high temperature thermal processes deliver a large thermal budget to the device, which is the biggest limitation for flexible and stretchable device applications. To overcome this barrier, many recent studies have proposed low temperature microwave annealing (MWA) technique using electromagnetic waves and the surface treatment technique using plasma in this regard. The MWA heats the sample through the absorption of electromagnetic waves, allowing a uniform heat energy transfer inside the device. A material absorbs microwave energy and converts it into heat, i.e., the heating pattern during microwave processing is inherently internal. Therefore, MWA demonstrates a high heat treatment effect in a short time because of the extremely high heat transfer efficiency resulting from the volumetric heating of the material^14–16^. MWA as calcination annealing of IGZO nanofibres is more efficient in removing the polymer matrix in the fibres because it is volumetric heating method. Furthermore, since plastic or glass substrates—which are transparent to microwaves—are not heated, it is a highly efficient heat treatment method that can selectively heat only IGZOs because they can easily absorb microwave energy^17^. In the case of *a*-IGZO films, a number of studies have been reported on the optimization of plasma treatment with argon (Ar) and oxygen (O_2_) gases^18–23^. The Ar-plasma treatment drastically improves the net electronic carrier concentration of *a*-IGZO thin films due to the oxygen-deficient IGZO stoichiometry without changing the composition of In, Ga, and Zn cations^22^. The O_2_-plasma treatment removes carbon-based impurities from *a*-IGZO thin films; hence, it greatly improves the device performance and reliability^23^. However, the plasma treatment studies on electrospun IGZO nanofibres have not been reported previously. Therefore, it is essential to establish an optimized processing conditions for the electrospun IGZO nanofibres for significant advances in device performance.

In this study, we investigated the effect of low thermal budget MWA for the calcination of electrospun IGZO nanofibres. In addition, the plasma surface treatment using various ratios (5 conditions) of Ar/O_2_ gas mixtures was performed to further improve the electrical properties of IGZO nanofibres. Scanning electron microscopy (SEM) and X-ray photoelectron spectroscopy (XPS) were used to analyse the surface morphology and chemical composition of the nanofibres pre- and post-plasma treatment, respectively. Then, we fabricated IGZO nanofibres FETs to investigate the effects of MWA calcination and Ar/O_2_ mixed-plasma surface treatment on the electrical properties and reliability of the nanofibres-based transistors. Subsequently, these nanofibres-based transistors were used in resistor-loaded inverters to evaluate the characteristics of IGZO nanofibres FET-based inverters. The instability tests using positive bias temperature stress (PBTS) and negative bias temperature stress (NBTS) were conducted to optimize the plasma treatment conditions.

## Results

### Morphological properties of electrospun IGZO nanofibres

**Figure 1:**
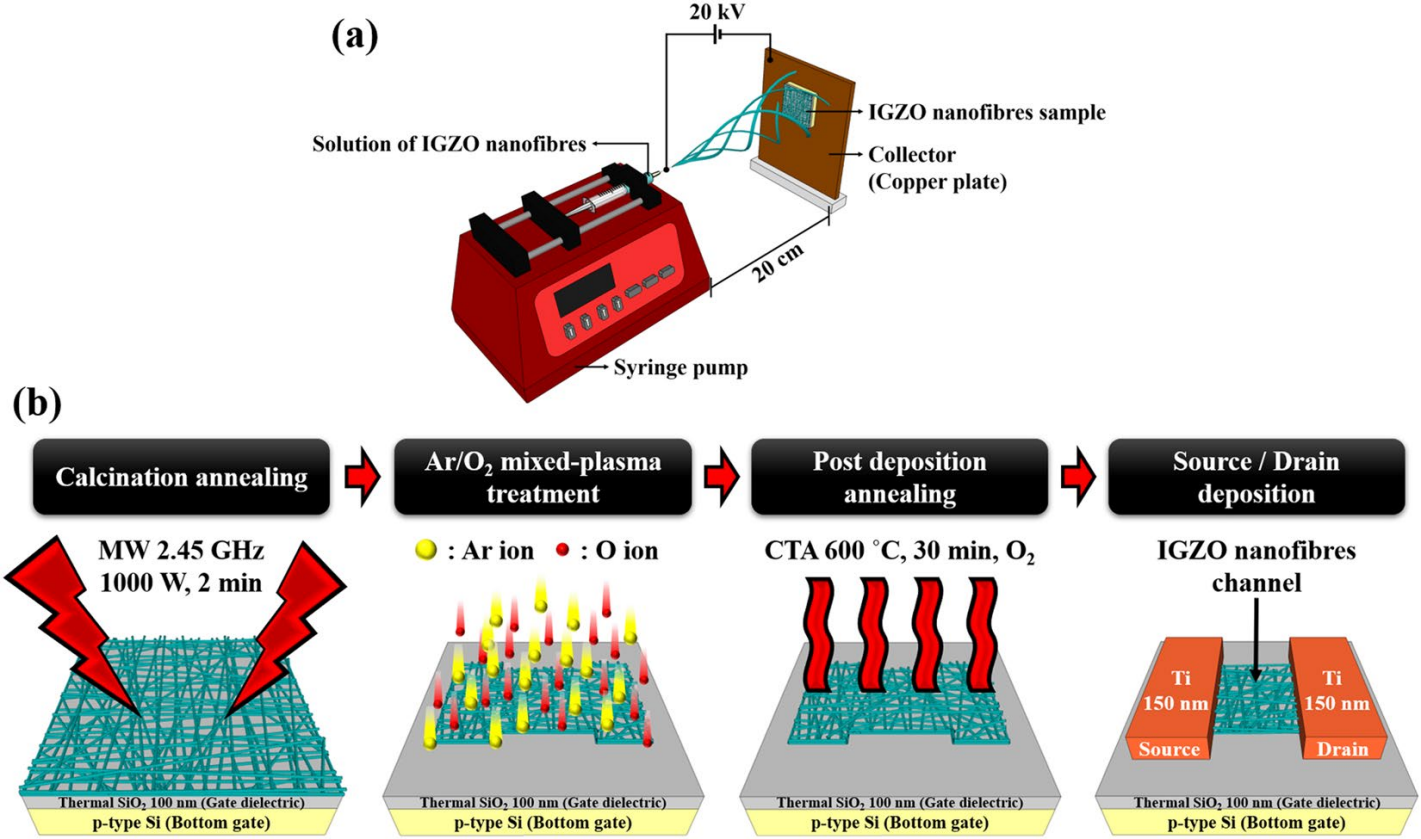
Schematic diagram of (**a**) the electrospinning apparatus and (**b**) step-by-step procedure for the fabrication of IGZO nanofibres FETs.

The experimental setup of the electrospinning apparatus is shown in Fig. 1a, which utilizes NE-1000 of New Era Pump System as the syringe pump. The syringe pump pressure was set as 0.2 ml/hr for electrospinning the IGZO nanofibres. A voltage of 20 kV was applied to the needle to initiate the jet, and a grounded square copper plate (15 × 15 cm^2^) was used as a collector for nanofibres. The temperature and humidity were maintained at 25 °C and 25%, respectively. The entire process took approximately 2–4 minutes to ensure an adequate amount of sample collection. Figure 1b shows a schematic diagram of the fabrication process of IGZO nanofibres FETs. IGZO nanofibres were electrospun on a *p*-type Si (100) substrate with a 100-nm-thick thermally grown SiO_2_ gate insulator layer. Then, the calcination of IGZO nanofibres was performed in ambient air for 2 min using a 1000 W microwave irradiation system with a frequency of 2.45 GHz. Subsequently, IGZO nanofibres were exposed to the plasmas of Ar/O_2_ gas mixture with different flow rates in sccm (50/0, 40/10, 25/25, 10/40, 0/50) for removing carbon-based impurities and improving their electrical properties.

**Figure 2:**
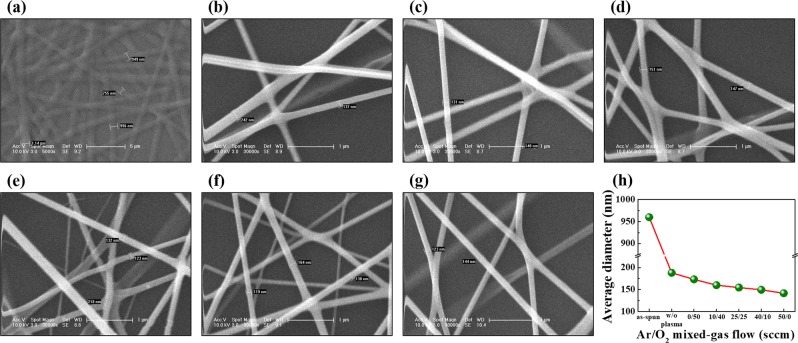
SEM images of IGZO nanofibres (**a**) as-spun at 5,000 times magnifications, (**b**) without plasma treatment, and with Ar/O_2_ mixed-plasma treatment under gas flow rates (in sccm) in the ratio of (**c**) 50/0, (**d**) 40/10, (e) 25/25, (**f**) 10/40, and (**g**) 0/50. (**h**) Average diameter of the electrospun IGZO nanofibres with plasma treatment at 30,000 times magnifications.

Figure 2 shows SEM images of as-electrospun and plasma treated IGZO nanofibres with the average diameter of the nanofibres after each process. The as-spun IGZO/polyvinylpyrrolidone (PVP) composite nanofibres display a uniform morphology (Fig. 2a) with an average diameter of approximately 960 nm. A remarkable decrease (~770 nm) in the diameter of nanofibres was observed after MWA calcination, as shown in Fig. 2b. The reduction in the diameter occurs due to the release of low molecular weight minerals, such as pyrrolidone, into the atmosphere when IGZO/PVP composite nanofibres are exposed to the microwave radiation. In addition, Ar/O_2_ mixed-plasma treated IGZO nanofibres show a further reduction in their diameter with an increase in the Ar ratio, as shown in Fig. 2c‒g. The average diameter of electrospun IGZO nanofibres is shown in Fig. 2h. The diameter of the IGZO nanofibres shown in Fig. 2h was averaged from 30 nanofibres selected from SEM images for each plasma treatment condition. The reduction in diameter is caused by the etching of the IGZO nanofibres by the additional Ar plasma, as Ar ions increase in the gas ratio of the Ar/O_2_ mixed-plasma treatment^24,25^.

### Electrical properties of IGZO nanofibres FETs

**Figure 3:**
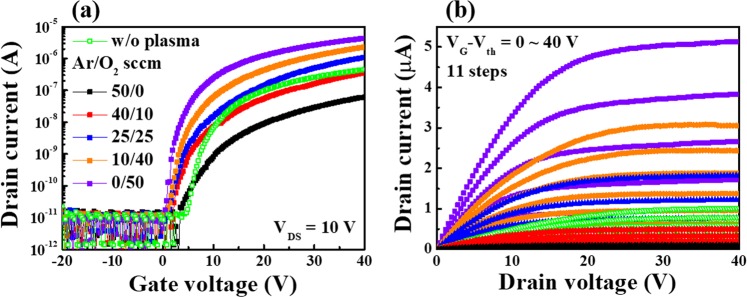
(**a**) Transfer curves and (**b**) Output curves of IGZO nanofibres FETs treated with various flow rate (in sccm) ratios of Ar/O_2_ mixed-plasma.

Since Ar and O_2_ plasma treatments may result in enhancement or degradation of the performance of IGZO nanofibres FETs depending on the treatment conditions, it is essential to establish the optimized plasma treatment conditions for maximizing the device performance^18–23^. Figure 3 illustrates (a) transfer curves (I_D_-V_G_) and (b) output curves (I_D_-V_D_) of IGZO nanofibres FETs treated with various ratios of Ar/O_2_ mixed-plasma. The device performance was found to be strongly dependent on the gas ratios of Ar/O_2_ mixed plasma. As the ratio of O_2_ in the Ar/O_2_ mixed-gas flow increases, the drain current increases. The maximum drain current is observed at the Ar/O_2_ flow rate (sccm) ratio of 0/50 (pure oxygen atmosphere). In contrast, the drain current of the nanofibres FET decreases with an increase in the Ar ratio of the Ar/O_2_ mixed-gas flow. For the Ar/O_2_ flow rate (sccm) of 40/10 and 50/0 (higher Ar content), the electrical properties of the device deteriorated compared with the ones without plasma treatment. The corresponding electrical parameters extracted from I_D_-V_G_ curves (Fig. 3) are summarized in Table 1. As the O_2_ flow rate increases in the Ar/O_2_ mixed-gas, an improvement in the on/off current ratio (I_on_/I_off_), the threshold voltage (V_th_), the field effect mobility (μ_FE_), the sub-threshold swing (S.S.), and the interface trap density (D_it_) is observed. Conversely, a deterioration of the electrical parameters is observed with an increase in the Ar flow rate. Therefore, our results indicate that the higher O_2_ gas content used in the Ar/O_2_ mixed-gas plasma treatment yields better electrical characteristics of the IGZO nanofibres FETs.

**Table 1:** Electrical parameters of IGZO nanofibres FETs for various Ar/O_2_ mixed-plasma flow rates.

Ar/O_2_ mixed-gas flow [sccm]	I_on_/I_off_	V_th_ [V]	μ_FE_ [cm^2^/V·s]	S.S [mV/dec]	D_it_ [cm^−2^]
50/0	0.06 × 10^6^	5.3	0.43	735.2	2.67 × 10^12^
40/10	0.36 × 10^6^	2.7	1.92	405.8	1.47 × 10^12^
25/25	1.10 × 10^6^	2.1	3.68	351.7	1.27 × 10^12^
10/40	2.42 × 10^6^	1.8	4.23	315.4	1.14 × 10^12^
0/50	4.35 × 10^6^	0.9	5.32	282.1	1.02 × 10^12^
w/o plasma	0.46 × 10^6^	5.6	0.82	346.6	1.26 × 10^12^

**Figure 4:**
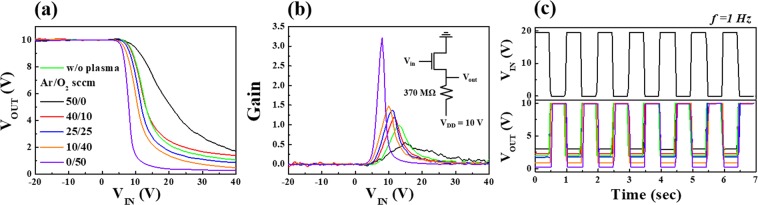
(**a**) Voltage transfer curves, (**b**) corresponding gain, and (**c**) dynamic responses of the resistor-loaded inverter based on IGZO nanofibres FETs fabricated after treatment with various flow rate (in sccm) ratios of Ar/O_2_ mixed-plasma.

After fabricating high performance electrospun IGZO nanofibres FETs through a combination of low thermal budget MWA calcination and Ar/O_2_ mixed-plasma surface treatment processes, we integrated the IGZO nanofibres FETs into resistor-loaded inverters. The typical voltage transfer curves (V_OUT_-V_IN_) of the resistor-loaded inverter based on IGZO nanofibres FETs are shown in Fig. 4a. Evidently, the output voltage (V_OUT_) of all inverters is inverted from the input voltage (V_IN_). The V_OUT_ level for the low V_IN_ was approximately equal to the supply voltage V_DD_ = 10 V. However, it should be noted that as the ratio of O_2_ in the Ar/O_2_ mixed-gas flow increases, the V_OUT_ level for the high V_IN_ approaches to 0 V. Figure 4b shows the corresponding gain (defined as -dV_out_/dV_in_) extracted from the V_OUT_-V_IN_ curves with the schematic of the circuit. The resistor-loaded inverter plasma-treated at Ar/O_2_ flow rate (sccm) ratio 0/50 (pure oxygen atmosphere) shows a maximum gain of 3.0; however, the gain decreases with increasing Ar ratio in the mixed-gas flow. Figure 4c shows the dynamic responses of the resistor-loaded inverter at V_DD_ = 10 V when V_IN_ is pulsed at 1 Hz. The inverter shows the closest behaviour (operation and response) to the square wave input signal under the mixed plasma flow rate (sccm) of Ar/O_2_ = 0/50. Therefore, we believe that the MWA calcination and Ar/O_2_ mixed-plasma treatment with low thermal budgets enhance the operational stability of IGZO nanofibres FETs.

### Reliability evaluation of IGZO nanofibres FETs

**Figure 5:**
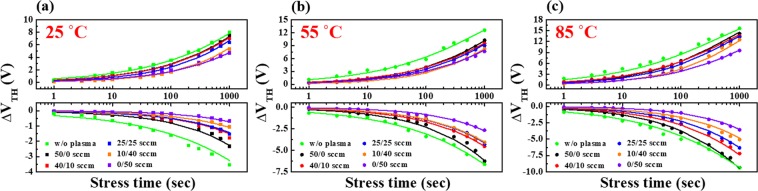
Time dependence of ΔV_th_ in PBTS and NBTS tests on various Ar/O_2_ mixed-plasma treated IGZO nanofibres FETs measured at (**a**) 25 °C, (**b**) 55 °C, and (**c**) 85 °C.

Although nanofibres structures have the advantages of being transparent, flexible, and stretchable, the extended use of nanofibres-based electronic devices can lead to instability of IGZO nanofibres FETs. In particular, the reliability of oxide semiconductor-based transistors is the most desired trait for practical applications^26,27^. To this end, several research groups have studied the reliability of IGZO TFTs; however, there has been no report on the reliability of IGZO nanofibres FETs in the literature. Therefore, we evaluated the electrical stability of the IGZO nanofibres FETs by observing the change in the threshold voltage (ΔV_th_) under prolonged gate bias stress and studied the effect of plasma treatment on the mixing ratio of Ar/O_2_. Figure 5 shows the ΔV_th_ of IGZO nanofibres FETs measured for 10^3^ seconds using PBTS (V_G_ = V_th0_ + 20 V) and NBTS (V_G_ = V_th0_ − 20 V) tests at 25, 55, and 85 °C. It is evident (from Fig. 5) that the threshold voltage V_th0_ of the non-plasma treated device shifts in the positive or negative direction depending on the polarity of the gate stress voltage. This is caused by the trapping of electrons or holes in the trap states, where ΔV_th_ measured during the PBTS and NBTS tests are affected by the oxygen related trap states (acceptor-like) and the oxygen vacancy related trap states (donor-like), respectively^26–28^. The Ar/O_2_ mixed-plasma treatment reduces the ΔV_th_ of the IGZO nanofibres FETs. In particular, a smaller value of ΔV_th_ was observed with an increase in the O_2_ gas ratio in the mixed-plasma. For each device, ΔV_th_ increases with the stress time and temperature, and the rate of increase is dependent on the stress temperature, as shown in Fig. 5a–c. A summary of measured ΔV_th_ after 10^3^ seconds of PBTS and NBTS tests for various Ar/O_2_ mixed-plasma treated IGZO nanofibres FETs at 25, 55, and 85 °C is shown in Table 2.

**Table 2:** ΔV_th_ after 10^3^ seconds of PBTS and NBTS tests for various Ar/O_2_ mixed-plasma treated IGZO nanofibres FETs measured at 25, 55, and 85 °C.

ΔV_th_ after 1000 s [V]							
Ar/O_2_ [sccm]		w/o plasma	50/0	40/10	25/25	10/40	0/50
PBTS @ 1000 s	25 °C	8.0	7.5	7.0	6.4	5.3	4.7
	55 °C	12.6	10.2	9.5	8.9	8.2	7.6
	85 °C	15.6	14.1	13.5	13.2	12.5	9.5
NBTS @ 1000 s	25 °C	−3.5	−2.3	−1.8	−1.5	−1.1	−0.7
	55 °C	−6.6	−6.3	−4.5	−4.3	−4.1	−2.6
	85 °C	−9.4	−9.3	−7.2	−6.3	−5.1	−3.6

The ΔV_th_ data in Fig. 5a–c was fitted using the following stretched-exponential equation for the charge trapping states^29^.

1 $$\Delta {V}_{th}(t)=\Delta {V}_{th0}[1-\exp \{-{(\frac{t}{\tau })}^{\beta }\}],$$

where ΔV_th0_ is the threshold voltage shift at an infinite time, τ is the characteristic trapping time of the charge carrier, and β is the stretched-exponential exponent. As shown in Fig. 5, the fitting curves are in good agreement with the experimental results, suggesting that τ depends on temperature, and the change in V_th_ occurs through the thermal activation process. In the stretched-exponential equation, the thermal activation of τ of electrons follows:

2 $$\tau ={\tau }_{0}\,\exp (\frac{{E}_{\tau }}{{k}_{B}T})={\nu }^{-1}\,\exp (\frac{{E}_{\tau }}{{k}_{B}T}),$$

where τ_0_ and *ν* are the thermal pre-factor and the frequency pre-factor for emission over the barrier, respectively. In addition, E_τ_ refers to the average effective energy barrier that must be overcome by the electrons in the IGZO nanofibres channel to be injected into the gate insulator, and it is related to the lattice arrangement of the IGZO nanofibres channel. A smaller E_τ_ means fewer defects or trap sites in the nanofibres channel, indicating a more ordered lattice arrangement^26^.

**Figure 6:**
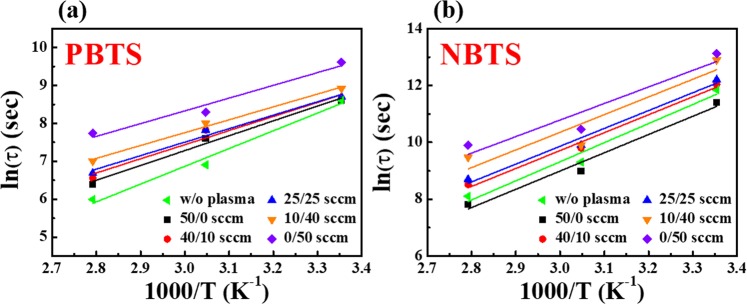
Characteristic trapping time (τ) under (**a**) PBTS and (**b**) NBTS tests as a function of reciprocal absolute temperatures for various Ar/O_2_ mixed-plasma treated IGZO nanofibres FETs.

In Fig. 6, the Arrhenius plots display the relationship between the logarithm of the characteristic trapping time ln (τ) and the inverse of the temperature 1/T. Figure 6a and b correspond to the PBTS and NBTS tests, respectively, for various Ar/O_2_ mixed-plasma treated IGZO nanofibres FETs. As evident from Fig. 6, the linear relationship between ln (τ) and 1/T indicates a thermally activated charge trapping process in these devices. The slope of the Arrhenius plot represents E_τ_ for the charge transport under the PBTS and NBTS tests. Table 3 summarizes the estimated values of E_τ_ under the PBTS and NBTS tests performed on the IGZO nanofibres FETs treated with various ratios of Ar/O_2_ mixed-plasma. Compared with the non-plasma treated device, the Ar/O_2_ mixed-plasma treated devices exhibited lower E_τ_. Particularly, a lower value of E_τ_ was observed for the devices treated with higher O_2_ gas ratio in the Ar/O_2_ mixed-gas flow. The smallest values of E_τ_ (~0.29 eV for PBTS and ~0.50 eV for NBTS) and the highest stability was obtained for the IGZO nanofibres channels treated with the Ar/O_2_ flow rate (sccm) ratio of 0/50 compared with other channels. These results are consistent with those displayed in Figs. 3 and 4.

**Table 3:** Average effective energy barrier (E_τ_) under the PBTS and NBTS tests of the IGZO nanofibres FETs treated with various ratios of Ar/O_2_ mixed-plasma.

Average effective energy barrier [eV]						
Ar/O_2_ [sccm]	w/o plasma	50/0	40/10	25/25	10/40	0/50
PBTS	0.40	0.34	0.32	0.31	0.29	0.29
NBTS	0.58	0.55	0.54	0.54	0.54	0.50

**Figure 7:**
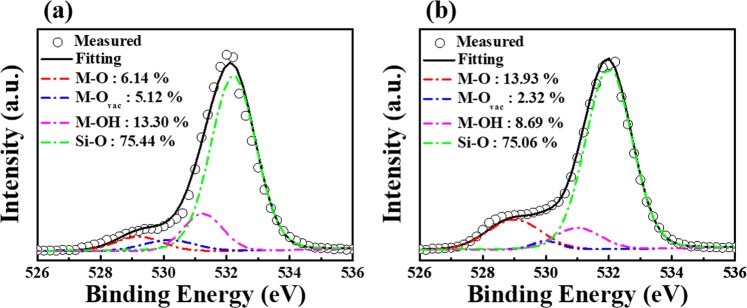
XPS O1s spectra for the Ar/O_2_ mixed-plasma treated IGZO nanofibres with gas flow rate (sccm) ratios of (**a**) 50/0 (purely Ar atmosphere) and (**b**) 0/50 (purely O_2_ atmosphere).

Figure 7 shows the XPS O1s spectra to evaluate the chemical states of IGZO nanofibres after the Ar/O_2_ mixed-plasma treatment with the flow rate (sccm) ratios of 50/0 (Fig. 7a) and 0/50 (Fig. 7b). The XPS spectra were collected to a depth of few-nanometres beyond the etched surface of IGZO nanofibres by Ar ions for avoiding any surface contamination. Unlike thin films of uniform thicknesses, the IGZO nanofibres have several gaps between the fibres as shown in Fig. 2; therefore, the components of the underlying SiO_2_ gate insulator are also observed in the XPS spectra. Thus, we have deconvoluted the O1s spectra into four individual component peaks. The peaks observed at 529, 530, and 531 eV are related to the components of the IGZO nanofibres, whereas the peaks at 532 eV is associated with the underlying SiO_2_ gate insulator. The main peak at 529 eV represents the stoichiometric oxygen (M-O), the subpeak at 530 eV represents the oxygen vacancies (M-O_vac_), and the subpeak at 531 eV is associated with the loosely bound oxygen impurities (M-OH) such as chemisorbed oxygen, H_2_O, and CO_3_. Since the Si-O peaks observed from the underlying SiO_2_ gate insulators show a similar contribution in both spectra, we focus our discussion only on the M-O, M-O_vac_, and M-OH bonds detected from the IGZO nanofibres. As evident from Fig. 7, the ratio of M-O_vac_ and M-OH bonds is smaller in the IGZO nanofibres treated with the Ar/O_2_ mixed-plasma at a flow rate (sccm) ratio of 0/50 (purely oxygen atmosphere) compared with the ones treated at 50/0 (purely Ar atmosphere). The oxygen vacancies of M-O_vac_ bonds are known to cause stability deterioration in gate bias temperature stress test^30^, whereas the loosely bound oxygen impurities of M-OH bonds act as the charge trap states and reduce the on-current in IGZO films^31^. Hence, we conclude that the mixed-plasma treated IGZO nanofibres FETs treated with Ar/O_2_ flow rate (sccm) ratio of 0/50 exhibit better electrical properties and reliability due to the low impurity concentration compared with those treated at 50/0 flow rate.

## Conclusion

In this study, we employed the low thermal budget MWA process for calcination of electrospun IGZO nanofibres and investigated the effects of Ar/O_2_ mixed-plasma surface treatment on the performance of IGZO nanofibres-based FETs. The MWA process (at 2.45 GHz and 1000 W) allowed rapid calcination (in 2 min) of the electrospun IGZO nanofibres in the ambient air. Additionally, the plasma surface treatment with Ar/O_2_ gas mixtures altered the surface morphology and chemical composition of the IGZO nanofibres, eventually affecting the electrical properties and reliability of the IGZO nanofibres FETs. Our results indicate that oxygen in the Ar/O_2_ mixed-plasma contributes to the removal of impurities, whereas argon plays a role in reducing the diameter of the IGZO nanofibres. In addition, the higher oxygen content in the Ar/O_2_ mixed-plasma significantly improved the performance of the IGZO nanofibres FETs and resistor-loaded inverters based on IGZO nanofibres FETs. Conversely, the higher argon content was found to degrade the performance of these devices. Instability tests using PBTS and NBTS revealed that O_2_ plasma treatment contributed to the stability improvement of the IGZO nanofibres FETs. Therefore, we expect that the MWA calcination process combined with the Ar/O_2_ mixed plasma surface treatment is a promising low thermal budget approach for the fabrication of high performance IGZO nanofibres FETs.

## Methods

### Solution synthesis procedure

Solution of IGZO nanofibres was synthesized by the sol-gel reaction of precursors. Indium nitrate hydrate [In(NO_3_)_3_·xH_2_O], gallium nitrate hydrate [Ga(NO_3_)_3_·xH_2_O], and zinc acetate dehydrate [Zn(CH_3_COO)_2_·2H_2_O] were used as precursors for the sol-gel reaction. These precursors were dissolved in N, N-dimethylformamide (DMF) solvent in a molar ratio of 2:1:1, and the solution was mixed at room temperature (25 °C) for 2 hours using a stirrer. Then, 2.5 ml of ethanol containing 0.18 g of polyvinylpyrrolidone (PVP, *M*_*W*_ ≈ 1300000) was added to the solution, and it was mixed at room temperature (25 °C) for 2 hours using a stirrer.

### Device fabrication procedure

A 100-nm SiO_2_ gate insulator is thermally grown and RCA cleaned on the p-type Si (100) substrate. Subsequently, a channel layer was formed by spinning IGZO nanofibres using the electrospinning method. To remove the polymer matrix, the MWA calcination annealing was performed by using a 1000 W microwave irradiation system with a frequency of 2.45 GHz in the ambient air for 2 min. Then, the active channel region of the IGZO nanofibres FETs was defined by photolithography and wet-etching technique using a 30:1 ratio of buffer oxide etchant (BOE). The width of the defined active channel region is 10 μm, and the length is 20 μm. Further, the IGZO nanofibres were exposed to the plasma of various Ar/O_2_ mixed-gas flow rates (in sccm; 50/0, 40/10, 25/25, 10/40, 0/50) in a reactive-ion etching (RIE) system. The power of the plasma treatment, the forward pressure, and the exposure time were selected as 200 W, 300 mTorr, and 20 s, respectively. To improve the electrical properties, the PDA process was performed, involving the conventional thermal annealing (CTA) in a furnace at 600 °C in O_2_-atmosphere for 30 min. Finally, IGZO nanofibres FETs were fabricated by depositing a 150 nm-thick Ti layer using e-beam evaporator and forming the source and drain electrodes of FETs through the lift-off process.

## Supplementary information

Supplementary information.
