# Supplementary material for: Performance improvement in electrospun InGaZnO nanofibres field-effect-transistors using low thermal budget microwave calcination and Ar/O2 mixed-plasma surface treatment
Source: Sci Rep. 2020 Feb 27;10:3645. doi: 10.1038/s41598-020-60637-8 (PMC7046654; doi:10.1038/s41598-020-60637-8)
Supplement: Supplementary file 1 — Supplementary information. [file 41598_2020_60637_MOESM1_ESM.docx]

Performance improvement in electrospun InGaZnO nanofibres field-effect-transistors using low thermal budget microwave calcination and Ar/O_2_ mixed-plasma surface treatment

Seong-Kun Cho and Won-Ju Cho^*^

Department of Electronic Materials Engineering, Kwangwoon Univ. Chambit-kwan, B 104, Wolgye 1-dong, Nowon-gu, Seoul 139-701, Korea

^*^E-mail: [chowj@kw.ac.kr](mailto:chowj@kw.ac.kr)

**Supplementary Information**

**1. Temperature of microwave annealing**

**Fig. S1** shows the temperature calibration of an infrared (IR) thermometer and the temperature measurement of samples in an MW system. **Initially,** as shown in Fig. S1a, we measured the sample temperature on the hot plate at various temperatures simultaneously using a thermocouple (TC) thermometer and an IR thermometer. **Next,** the temperature measured by the TC thermometer and the temperature measured by the IR thermometer were compared. **Fig. S1b** shows the temperature (open black squares) of a sample measured with a TC thermometer and an IR thermometer. It turns out that there is a difference between the TC thermometer, which indicates the actual sample temperature, and the IR thermometer. As the sample temperature increased, the difference (open blue triangles) gradually increased, and therefore, it was necessary to correct the IR thermometer. The IR thermometer was calibrated using the correlation between these temperatures. After calibration, the IR thermometer and the TC thermometer can be seen to be in good agreement (closed red circles). Finally, we installed a calibrated IR thermometer in the microwave system to monitor the temperature of the sample in real time as shown in **Fig. S1c**.


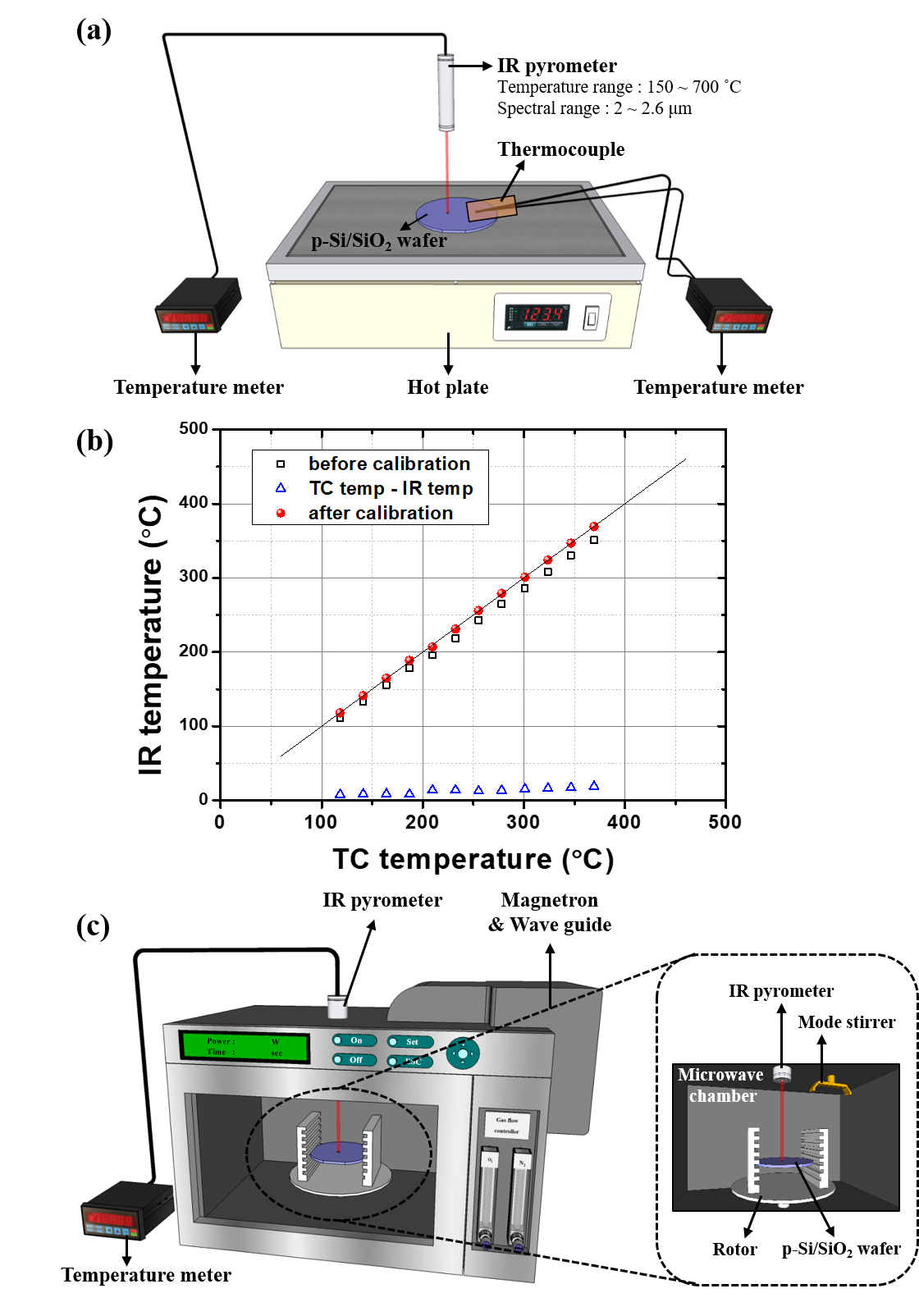


**Figure S1.** Temperature calibration of infrared (IR) thermometers and sample temperature measurement in MW systems: (a) simultaneous measurement of sample temperature on hot plate using TC and IR thermometers, (b) sample temperature measured simultaneously with TC and IR thermometers, and (c) microwave annealing system with calibrated IR thermometer.

**Fig. S2a** illustrates the temperature of an IGZO nanofibre sample according to microwave power measured using the IR thermometer. IGZO nanofibre samples were prepared by electrospinning IGZO nanofibres on a p-Si/SiO_2_ substrate. The inset shows the temperature of the sample with respect to the microwave irradiation time. It can be seen that within 20 seconds the temperature is nearly saturated and then remains almost constant regardless of the irradiation time. The temperature of the IGZO nanofibre samples increased linearly with increasing microwave power, reaching approximately 530 °C at 1800 W, which is the highest temperature available in our microwave irradiation system. In addition, the microwave power of 1000 W applied in this experiment corresponds to 430 °C.

**Fig. S2b** shows the difference in temperature of p-Si/SiO_2_ substrate with and without IGZO nanofibres irradiated with a microwave power of 1000 W. Depending on the type of material, microwaves have the property of reflecting, transmitting or absorbing.^S1^ Metals reflect microwaves, while transparent materials are classified as insulators. Materials which are transparent to microwaves are classed as insulators. Materials which are excellent absorbers of microwave energy are easily heated and are classed as dielectrics.

Since SiO_2_ is almost transparent to microwaves, it is selectively absorbed and only generates heat mainly by Si and IGZO nanofibres. The heat generated by microwaves is determined by the dielectric constant of the dielectric material, and the dielectric constants of Si and IGZO are similar, 11.9 and 10, respectively. Therefore, as shown in **Fig. S2b**, there is no significant difference in temperature on the p-Si/SiO_2_ substrate with or without IGZO nanofibres.


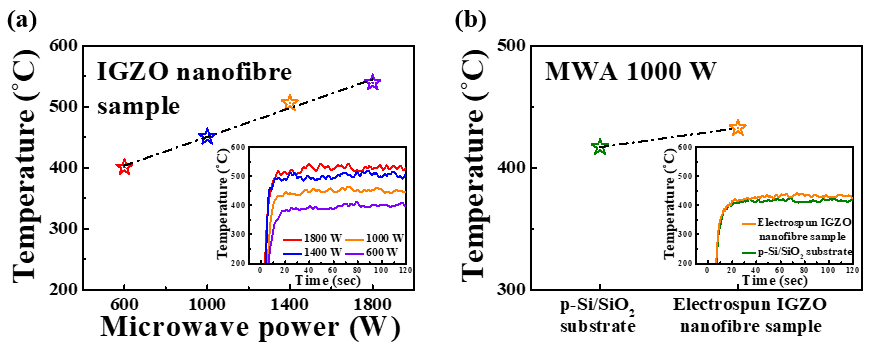


**Figure S2.** (a) Temperature of the electrospun IGZO nanofibre samples according to the microwave irradiation power measured by an IR thermometer. (b) Temperature at 1000 W microwave power of p-Si/SiO_2_ substrates and electrospun IGZO nanofibre samples.

**2. Electrical properties of IGZO nanofibres FETs**

In this study, microwave annealing (MWA) was applied for calcination annealing on electrospun IGZO nanofibres, followed by conventional thermal annealing (CTA) in a resistive furnace for post-deposition annealing.

**Fig. S3** shows the transfer curves (I_D_-V_G_) of IGZO nanofibres field-effect-transistors (FETs). From the transfer curves shown in **Fig. S3a**, we can see that the electrical properties improve with microwave power, but are nearly saturated above 1000 W. Therefore, from the viewpoint of energy consumption, it is considered that the micropower of 1000 W is suitable. In the case of irradiation time, the electrical properties were almost saturated at 2 minutes, so MWA was performed based on the irradiation time of 2 minutes.

As a result of evaluating the characteristics according to the microwave annealing ambient, as shown in **Fig. S3b**, the performances of the IGZO nanofibres FETs were poor in the N_2_, but excellent in the O_2_ and air. It is found that pure O_2_ treatment can achieve the electrical properties of IGZO nanofibres FETs that are much better than pure N_2_ treatment. On the other hand, air ambient is much better than pure N_2_, and can obtain almost equivalent electrical properties as pure O_2_. Therefore, we applied air ambient annealing for low cost processes.

In addition, to evaluate the difference according to the calcination annealing method, the electrical properties of IGZO nanofibres FETs with MWA or CTA calcination annealing and without calcination annealing were compared as shown in **Fig. S3c**. The IGZO nanofibres FETs without calcination annealing have very poor transfer characteristics, indicating that impurities have not been sufficiently removed from the nanofibres. Furthermore, to compare the two processes as calcination annealing, the CTA was performed by annealing at 600 °C for 30 minutes in O_2_ ambient, whereas MWA was performed at 1000 W for 2 minutes in air ambient. We have confirmed in advance that these conditions are optimal for each process. The results show that calcination annealing is essential, and that MWA calcination has better electrical properties than CTA calcination despite much lower temperatures and shorter process times. Thus, it can be concluded that MWA calcination annealing can significantly reduce the thermal budget delivered to the device and further improve the electrical properties.

**Fig. S3d** shows the electrical properties of IGZO nanofibres FETs with CTA temperature to establish optimal PDA conditions. The calcination annealing for these devices were performed by MWA at 1000 W for 2 minutes in air ambient. As the temperature of the CTA increases, the drain current of the IGZO nanofibres FETs increases, improving device performance. Therefore, we determined a CTA of 600 °C as the optimal PDA condition and applied it to this study.


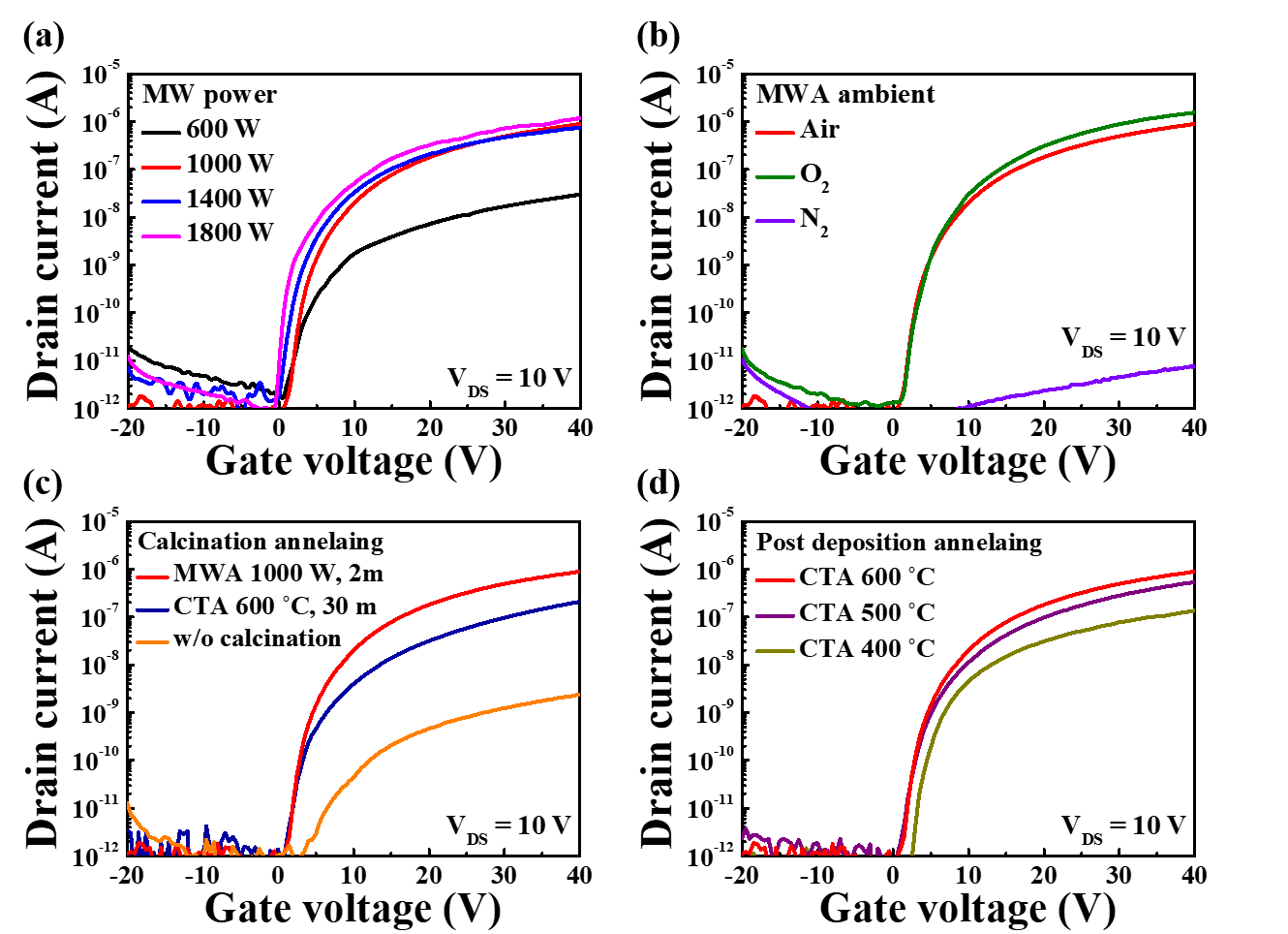


**Figure S3.** Transfer characteristic curves of IGZO nanofibres FETs according to (a) power of microwave irradiation system in air ambient, (b) annealing ambient of air, O_2_, and N_2_ at 1000 W microwave power, (c) calcination annealing method, and (d) temperature of PDA.

**Fig. S4** shows the transfer curves of the IGZO nanofibres FETs for the plasma processing sequence after MWA and the effect of CTA as post-plasma processing. Ar/O_2_ mixed-gas flow rate, power, process pressure and exposure time in the plasma treatment were set to 25/25 sccm, 200 W, 300 mTorr, and 20 s, respectively. The device subjected to CTA treatment after plasma treatment (red solid line) has significantly improved electrical characteristics than the plasma treatment after CTA treatment (black dot line). In particular, the device subjected to plasma treatment after CTA (black dot line) has worse characteristics than the device without plasma treatment (blue dash-dot line), and it can be inferred that characteristic deterioration by plasma occurs. These results indicate that plasma treatment affects IGZO nanofibres channels or SiO_2_ gate insulators exposed to plasma, but can be recovered through additional CTA.


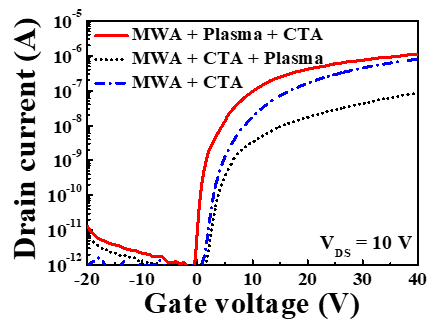


**Figure S4.** Transfer characteristic curves of the IGZO nanofibres FETs for the plasma processing sequence after MWA and the effect of CTA as post-plasma processing.

**3. Contact resistance of IGZO nanofibres FETs**

We extracted the contact resistance of S/D electrode according to various Ar/O_2_ plasma conditions using transmission line method (TLM) pattern. **Fig. S5a** shows the total resistance of IGZO nanofibres FETs versus channel length at a gate voltage of 40 V. At large gate voltages, the channel resistance decreases, and the contact resistance can be obtained from the intercept on the Y axis. ^S2^ **Fig. S5b** shows the contact resistance extracted by the TLM. As the ratio of O_2_ in the Ar/O_2_ mixed gas flow increases, the contact resistance decreases. The minimum contact resistance is observed at an Ar/O_2_ flow rate (sccm) ratio of 0/50 (pure oxygen ambient). On the other hand, as the Ar ratio increases, the contact resistance increases, resulting in a higher contact resistance for Ar/O_2_ flow rates of 40/10 and 50/0 (pure argon ambient) than without plasma treatment.


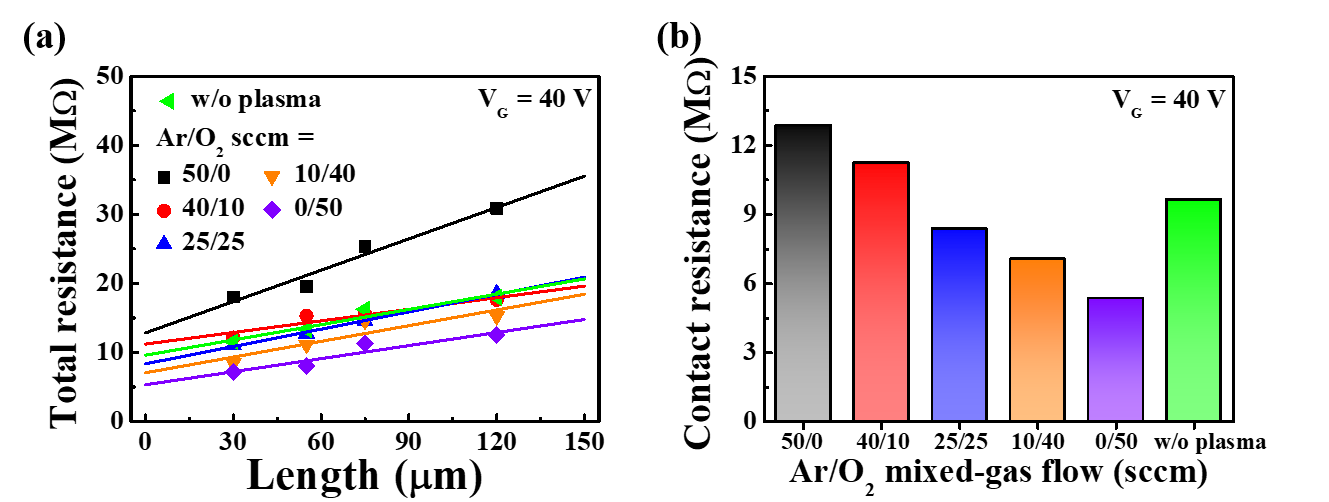


**Figure S5.** (a) Total resistance as a function of FET channel length, and (b) contact resistance for various Ar/O_2_ mixed-gas flow.

**4. References**

1. Haque, K. E. Microwave energy for mineral treatment processes—a brief review. International journal of mineral processing, **57**, 1-24 (1999).
2. Kim, W. S., Moon, Y. K., Kim, K. T., Lee, J. H., & Park, J. W. An investigation of contact resistance between metal electrodes and amorphous gallium–indium–zinc oxide (a-GIZO) thin-film transistors. Thin Solid Films, **518**, 6357-6360 (2010).
